# Climate Change and Food Security: Health Impacts in Developed Countries

**DOI:** 10.1289/ehp.1104424

**Published:** 2012-06-27

**Authors:** Iain R. Lake, Lee Hooper, Asmaa Abdelhamid, Graham Bentham, Alistair B.A. Boxall, Alizon Draper, Susan Fairweather-Tait, Mike Hulme, Paul R. Hunter, Gordon Nichols, Keith W. Waldron

**Affiliations:** 1 School of Environmental Sciences, and; 2 Norwich Medical School, University of East Anglia, Norwich, United Kingdom; 3 Environment Department, University of York, York, United Kingdom; 4 Centre for Public Health Nutrition, University of Westminster, London, United Kingdom; 5 Centre for Infections, Health Protection Agency, London, United Kingdom; 6 Institute of Food Research, Norwich, United Kingdom

**Keywords:** adaptation, climate change, food safety, food security, nutrition, regulation

## Abstract

Background: Anthropogenic climate change will affect global food production, with uncertain consequences for human health in developed countries.

Objectives: We investigated the potential impact of climate change on food security (nutrition and food safety) and the implications for human health in developed countries.

Methods: Expert input and structured literature searches were conducted and synthesized to produce overall assessments of the likely impacts of climate change on global food production and recommendations for future research and policy changes.

Results: Increasing food prices may lower the nutritional quality of dietary intakes, exacerbate obesity, and amplify health inequalities. Altered conditions for food production may result in emerging pathogens, new crop and livestock species, and altered use of pesticides and veterinary medicines, and affect the main transfer mechanisms through which contaminants move from the environment into food. All these have implications for food safety and the nutritional content of food. Climate change mitigation may increase consumption of foods whose production reduces greenhouse gas emissions. Impacts may include reduced red meat consumption (with positive effects on saturated fat, but negative impacts on zinc and iron intake) and reduced winter fruit and vegetable consumption. Developed countries have complex structures in place that may be used to adapt to the food safety consequences of climate change, although their effectiveness will vary between countries, and the ability to respond to nutritional challenges is less certain.

Conclusions: Climate change will have notable impacts upon nutrition and food safety in developed countries, but further research is necessary to accurately quantify these impacts. Uncertainty about future impacts, coupled with evidence that climate change may lead to more variable food quality, emphasizes the need to maintain and strengthen existing structures and policies to regulate food production, monitor food quality and safety, and respond to nutritional and safety issues that arise.

There is widespread agreement that anthropogenic greenhouse gas (GHG) emissions are leading to climate change (Stern 2006). This will have a number of impacts, which will include changes in food production and supply (Lobell et al. 2011; Strategy Unit 2008). In the literature, there is much focus on the effects of climate change on food security (defined as access to sufficient, safe, nutritious food to maintain an active and healthy lifestyle) in the developing world [World Health Organization (WHO) 2010]. In these developing areas, there is good evidence that climate change will compound existing and predicted food insecurity and undernutrition (Cohen et al. 2008). For example, by the end of this century, the average summer temperature will exceed the hottest summer on record throughout the tropics and subtropics, with potentially serious consequences for food production that could affect the 50% of the world’s population living in such regions (Battisti and Naylor 2009). However, in developed countries, food shortages are uncommon and shortage of energy is not a major problem, although micronutrient deficiencies and overnutrition are prevalent. The nutritional quality and safety of food are the primary concerns related to food in these areas.

Climate change is likely to have a number of consequences for food security in developed countries, and these effects are enacted through multiple pathways, as summarized in Figure 1. Anthropogenic GHG emissions and natural climate forcings (other mechanisms that lead to climate variability such as stratospheric volcanic aerosols) (Hegerl et al. 2011) lead to climate change and specific environmental effects, which have an impact on agriculture and food processing. The agrifood industry will respond to a changing climate (adaptation) and will be affected by initiatives to modify farming and food systems to reduce GHG emissions associated with the food chain (mitigation) (Royal Society 2009). They may also become involved in further initiatives to reduce GHG emissions through the production of biofuels (Banse 2008). All these will lead to changes in the types of food that individuals consume, their nutritional content and safety. Climate change will also directly influence food choice. Finally, as mitigation against climate change, there may be increased uptake of low GHG diets (preferentially consuming food whose production, processing, storage and transportation releases lower GHG emissions). Any changes to food choice or the conditions under which food is produced may have consequences for the nutritional composition of diets and food safety, hence important impacts on health (Royal Society 2009).

**Figure 1:**
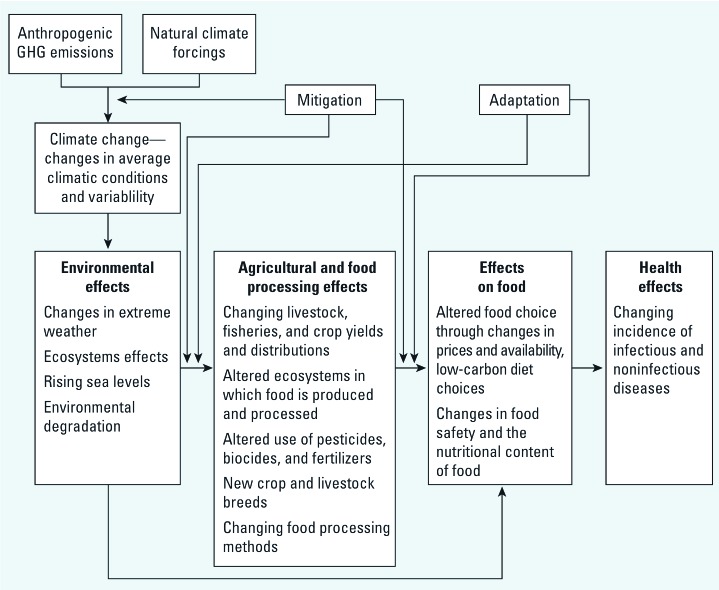
Main pathways through which climate change affects food security in developed countries (adapted from McMichael et al. 2006). Natural climate forcings are nonanthropogenic mechanisms that affect climate, such as stratospheric volcanic aerosols. The causes and main impacts of climate change are shown on the left. GHG, greenhouse gases.

To investigate the potential impacts of anthropogenic climate change upon food security, it is important to recognize that food is a global commodity. Food consumed in one part of the world may be produced thousands of kilometers away. Countries also buy food on an international market, so changes in food production in one part of the world may affect the price of food produced in other parts of the world.

This review aimed to investigate the possible impact that anthropogenic climate change may have on nutrition and food safety and on the subsequent consequences for health in developed countries. The existing literature includes much research on climate change and agriculture but less on other components such as climate change and nutrition. We focused specifically on the effects of climate change upon food in developed countries using the United Kingdom as a case study. We first consider how climate change may affect the nutritional composition and safety of food that individuals consume. We then consider the ability of developed countries to adapt to climate change, specifically looking at the complex policies and structures (e.g., legislation) in place to regulate food production, monitor the quality and safety of food, and record and respond to any health consequences associated with its consumption.

This review was not a formal systematic review due to the breadth of the topic. Instead, we began by conducting interviews with eight of the authors (G.B., A.B.A.B., A.D., S.F.-T., M.H., P.R.H., G.N., and K.W.W.) who were chosen for their knowledge of a range of issues related to climate change and food security. The aim of these interviews was to ascertain how climate change might interact with food and then to identify the main research projects and key papers dealing with these issues. This allowed us to set out the main structure of the review. The results of the interviews were used to begin to identify the main issues to be explored, in conjunction with a broad-based literature review using Google (2009). To fill in important gaps, we carried out specific focused searches in additional databases [including MEDLINE (National Library of Medicine 2009), Embase (2009), ISI Web of Knowledge (2009), and SCOPUS (2009)] to find key references. Initially, the search focused on reviews in the relevant areas published in the peer-reviewed and gray literature. These were then supplemented through specific searches for additional relevant primary and secondary research. The results were summarized, with established answers and remaining questions highlighted. The first draft of the review summarizing the work was sent to the eight experts for comments. These comments were incorporated into the review and further searching of the literature conducted if required. Finally, the review was evaluated by experts from the U.K. Food Standards Agency (FSA).

Much of the United Kingdom’s food is produced in Europe. Projections for this region suggest that climate change will result in warming of 2.1–4.4°C by 2080, with the greatest temperature increases occurring in northern and eastern Europe [European Environment Agency (EEA) 2007)]. Warming may be greatest during the winter in northern Europe and during the summer in southern and central Europe with increases of up to 6°C. Northern and eastern Europe are projected to become wetter, while the Mediterranean is expected to become drier. Projections about extreme events are highly uncertain, but heat waves are expected to be more intense, frequent, and longer lasting, whereas extreme precipitation events will increase in northern and western Europe (EEA 2007). In the United Kingdom, all areas are expected to become warmer, particularly in summer (United Kingdom Climate Impacts Programme 2011). Annual precipitation is not expected to change much overall, but patterns of precipitation are estimated to result in drier summers and wetter winters. Extreme precipitation and also heat events will become more common.

## Impacts of Climate Change

*Food prices and availability*. Several studies have examined the likely impact of climate change on world food prices, mostly of grain. As reviewed by Easterling et al. (2007), these studies suggest little change, or a small reduction, in grain prices up to a rise in global temperatures of 3°C after which prices will start to rise as production falls. However, many assessments do not consider likely increases in the frequency of extreme weather events predicted under climate change [Intergovernmental Panel on Climate Change (IPCC) 2007]. When these assessments are considered, Easterling et al. (2007) concluded that crop prices are likely to be higher than the published assessments. One example of the impact of current climate variability occurred in 2006 when extreme weather in many parts of the world, particularly the Murray–Darling Basin in Australia, led to reductions in world cereal production. These yield reductions were partly to blame for rising global food prices (Piesse and Thirtle 2009). Another example was the 25% reduction in the French fruit harvest after the 2003 European heat wave. Although extreme weather events have the potential to lead to localized food shortages, in the 2003 European heat wave the global food trade helped to avert regional food availability issues (Battisti and Naylor 2009).

One mitigation measure to combat climate change is increased use of biofuels, which, by displacing food crops from agricultural land, could lead to increased food prices. Biofuels have been implicated as one cause of the 2007 rise in global food prices (Lock et al. 2009, and it has been suggested that the European Union (EU) Biofuels Directive could slow down or reverse the long-term trend of declining world food prices (Banse et al. 2008). The production of biofuels in many countries is driven by policy measures such as tax exemptions, investment subsidies, and obligatory blending of biofuels with mineral fuels (Banse et al. 2008). Therefore, the future impact of biofuels will depend heavily upon how these policy measures are applied. Furthermore, technological changes, such as the development of second generation biofuels, that may have lower impacts upon existing agriculture, will also play a key role.

If rises in food prices occur, then individuals may shift to lower cost food items, which in turn, may have health consequences. During the recent increases in food prices, there is evidence from Scotland that consumers shifted from more expensive to cheaper brands of food, and away from organic produce (Rural and Environment Analytical Services 2009). Shifts from more expensive brands of food to cheaper ones may have few or may even have positive nutritional effects. For example, some cheaper brands of food have lower sodium and fat contents (Cooper and Nelson 2003). Movements away from organic produce are expected to have few, if any, nutritional consequences (Dangour et al. 2009). However, other shifts may be of more concern as healthier food is often more expensive than less healthy food (e.g., lean meat compared with fatty meat), and so rising prices often result in less healthy food choices (Cummins and Macintyre 2006). Of particular concern are energy-dense foods (usually more processed food with high-sugar and high-fat contents), which are often cheaper than their less energy-dense counterparts. Energy-dense foods are also less affected by increases in the costs of agricultural commodities because processing and marketing are major components of their cost [Economic and Social Research Council (ESRC) 2008]. Consequently, Lock et al. (2009) concluded that during recent food price increases, major fast food companies have seen large profits despite overall reductions in consumer spending. Therefore, these rises in food prices associated with climate change may reduce the nutritional quality of dietary intakes and lower the nutritional status of some groups. Rising prices could also increase the risk of obesity particularly among children, young adults, smokers, lower-income groups, and frail older people who already have more marginal nutritional status [Scientific Advisory Committee on Nutrition (SACN) 2006] and are more likely to be affected by rising prices. Such price rises raise equity concerns and are likely to exacerbate health inequalities (Lock et al. 2009).

Changes in food consumption because of increasing costs driven by climate change may also affect food safety as different foods carry varying risks of foodborne illness (Adak et al. 2005) and different levels of pesticide and chemical residues. For example, as the cost of food increases, consumers may shift from more expensive fresh poultry to frozen poultry, which may increase the likelihood of consuming chicken contaminated with *Salmonella*, but reduce the likelihood of consuming chicken contaminated with *Campylobacter* (FSA 2009a). In the absence of detailed information on likely shifts in purchasing and diet, it is difficult to predict changes in food safety or nutrition.

*Changing production methods*. With climate change, food will be produced under different climatic conditions in altered ecosystems, which will alter agricultural conditions and be compounded by adaptations to such change. Conditions may be further altered through initiatives from the food industry to mitigate against climate change. The food sector is a significant source of GHG emissions and food production, processing, transport, storage, preparation, purchase, and consumption that contributes 15–30% of global GHG emissions (Garnett 2008). Most GHG emissions arising from the food sector occur within agriculture (45%), food manufacture (12%), and transport (12%) (Garnett 2008). GHG-mitigation initiatives might include introducing high-sugar grasses into the diet of cows, which reduces methane emissions [Department for Environment, Food and Rural Affairs (Defra) 2011b], or altering the times of year when animal manures are spread onto land to reduce emissions of nitrous oxide (a GHG; Agricultural Land Advisory Service (ADAS 2009). These changes could have implications for nutritional quality and food safety.

Climate change may alter the seasonal patterns and abundance of pests and diseases, which may affect pesticide use, including herbicides and fungicides (Boxall et al. 2009). Responses will differ between crops and between geographical locations. For example, Chen and McCarl (2001) estimated that pesticide use in the United States would increase under climate change overall. However, the projected effects varied by crop and location, such that pesticide use on wheat was predicted to increase by 14% in Kansas but decrease by 10% in Colorado; pesticide use in Illinois was predicted to increase by 18% on corn but only by 3% on soya beans. Elevated temperatures may also lead to the emergence and re-emergence of pathogens, vectors, or hosts (Harrus and Baneth 2005), resulting in greater use of biocides and veterinary medicines in livestock management (Kemper 2008). This could increase the prevalence of antibiotic-resistant pathogens in animal and human populations [Food and Agriculture Organization (FAO) 2008].

Climate change could affect existing pathogens or lead to the emergence of new pathogens in food (Tirado et al. 2010), through effects on animal husbandry and animal-to-animal transmission, pathogen survival, and other mechanisms. Previous research has demonstrated that *Salmonella* infections in humans are positively associated with temperature (Kovats et al. 2004). This is biologically plausible because *Salmonella* bacteria will reproduce in food that is kept at ambient temperature. Therefore, under a warmer climate, elevated cases of salmonellosis are likely. However, for many other pathogens, although associations between human cases and weather exist (e.g., *Campylobacter* and temperature; Kovats et al. 2005), the biological mechanisms underpinning these associations are not fully understood, which makes it difficult to predict the effects of climate change. The pathogens most likely to be affected by climate change are those with low-infective doses (e.g., *Escherichia coli* strains and parasitic protozoa) where small changes in distribution or abundance could lead to many more human cases. Other pathogens likely to be affected are those with significant persistence in the environment (e.g. enteric viruses and parasitic protozoa) (FAO 2008). Pathogens with good stress tolerance responses to temperature and pH (e.g., *E. coli* and *Salmonella*) may also compete better against other pathogens under climate change (FAO 2008).

Agricultural adaptation to climate change may involve increased use of irrigation water. Döll (2002) estimated that climate change will lead to a 5–8% increase in crop irrigation requirements globally and increases as high as 15% in Southeast Asia. The use of wastewater for irrigation would reduce water extraction but could increase pathogen risks for consumers [World Health Organization (WHO) 2006]. For example, the 2008 *Salmonella* serotype Saintpaul outbreak in the United States, in which 1,500 people were allegedly infected, was linked to produce irrigated with wastewater in Mexico (Jungk et al. 2008). Elevated use of irrigation could also introduce chemicals into the food chain as such water may be contaminated with chemicals, such as pesticide residues (Boxall et al. 2009).

Agricultural adaptation to and mitigation against climate change will lead to the development of new crops and livestock species bred or engineered to survive in different climatic conditions or emit less GHGs. It will be important to monitor these new commodities to ensure that nutritional quality is maintained. For example, in the United Kingdom, a study using data from a long-term wheat-farming experiment found that since the mid-1960s the goal of increased food production was achieved at the expense of lower levels of zinc, iron, copper and magnesium in wheat (Fan et al. 2008).

Climate change may affect the transport of pathogens and chemicals into food. Examples of transfer mechanisms that may increase under climate change include aerial inputs of volatile and dust-associated contamination, flooding, and increased bioavailability of heavy metals due to changing environments and soil properties (Boxall et al. 2009). Climate change may alter the nature of the material being transported, as well as increasing the rates of transport. For example, after hurricanes Katrina and Rita, the U.S. Geological Survey found evidence that some mobilized flood sediments were derived from old, highly contaminated urban soils (Plumlee et al. 2007).

Climate change can affect food during its journey from the farm to consumer, and elevated temperatures may lead to increased bacterial replication (e.g., *Salmonella*) elevating food risks (Lake et al. 2009). Mycotoxins, an important public health concern, are formed through complex interactions between fungi and crops and are affected by weather and soil (Russell et al. 2010). A recent review indicated increasing problems of mycotoxins in parts of temperate Europe and the United States as climate change–associated temperature rises approach the optimal level for production of aflatoxins—one of the most important mycotoxins from a public health point of view. In other countries such as Australia, temperatures may rise to levels high enough to reduce fungal growth and mycotoxin production (Russell et al. 2010).

*Shifts to low-GHG diets*. Climate change may increase the consumption of lower GHG diets as a mitigation strategy. Fifty percent of European food-associated GHG emissions are due to meat and dairy consumption. These figures incorporate emissions from food production, processing, and distribution (Barrett et al. 2002; Wallén et al. 2004). Analysis of individual foods indicates that the consumption of meat and dairy foods, especially beef, lamb, pork, and cheese result in 3–13 times more GHG emissions than do vegetables and pulses (e.g., legumes) per unit weight (Wallén et al. 2004); this finding was confirmed by a Defra (2011a) study that collated evidence of environmental sustainability of foods based on the FSA’s Eatwell Plate. Shifts to low-GHG diets would reduce meat and dairy consumption, resulting in public health benefits and risks. Although a recent U.K. study estimated that a 30% reduction in red meat consumption would reduce ischemic heart disease by 15% (Friel et al. 2009), reductions in red meat consumption also may lower the iron and zinc nutritional statuses of certain vulnerable groups (SACN 2010). For example, the WHO (2008) estimated that in Europe 22% of preschool children, 25% of pregnant women, and 19% of nonpregnant women already have anemia. Such reductions in red meat consumption also may have food safety implications. Substituting meat with poultry or seafood might increase foodborne illnesses, whereas replacement with pulses and vegetables would reduce them (Adak et al. 2005).

Other foods associated with moderately large GHG emissions include sugary foods and drinks, tomatoes, peppers, rice, eggs, poultry, bagged salads, cooking oils, biscuits, and crackers (Wallén et al. 2004). A GHG-mitigation strategy that led to reduced consumption of sugary foods and drinks may be beneficial to oral health, but reduced consumption of tomatoes, peppers, and salads may be less beneficial. The overall nutritional and food safety implications of such shifts are difficult to judge without information on what these products would be replaced with.

Consuming food that is in season tends to lower GHG emissions. This is because out-of-season food production has greater agricultural inputs, such as the use of heated greenhouses, and hence GHG emissions (Garnett 2006). If low-GHG diets lead to reduced consumption of nonseasonal produce, this could adversely affect fruit and vegetable consumption in the winter and spring when local availability is limited in temperate countries. Ensuring adequate year-round consumption of a variety of fruit and vegetables is important for public health (WHO 1990) and has been one of the major beneficial changes in the diets of individuals over the past 40 years (Foster and Lunn 2007). Transport of food from other parts of the world where it is in season would be one solution to this problem, as would be storing seasonally produced food for other times of the year. These two options may be similar in terms of GHG emissions. One study suggested little difference in GHG emissions between consuming European-grown apples in the spring (harvested in the autumn and stored through the winter) and consuming imported apples from New Zealand (harvested in the European spring and shipped directly to Europe) (Blanke and Burdick 2005).

Consuming food that has traveled less distance (i.e., low food miles or local food) is a popular consumer concept, partly due to climate change concerns (Smith et al. 2005). However, for many foods, transport contributes only a small proportion to total GHG emissions (~12%; Garnett 2008). Therefore, a locally sourced diet is not necessarily a low-GHG diet. The exception, where transport is a large proportion of GHG emissions, is air-freighted food. In the United Kingdom, although only 1.5% of fruit and vegetables are air freighted, this accounts for 40% of fruit and vegetable GHG emissions associated with transport. Air-frieght transport is increasing at 6% each year (Garnett 2006), but it should be recognized that air-freighting may be beneficial to farmers in developing countries (MacGregor and Vorley 2006). If individuals change to a locally sourced diet to mitigate against climate change, then they are likely to find it difficult to achieve a year-round supply of fresh fruit and vegetables.

Consuming food from a small geographical area may also increase the risk of nutrient deficiencies or toxic effects reflecting the chemical characteristics of local soils (Oliver 1997). For example, one reason for the reduction of goiter (due to iodine deficiency) in the United Kingdom during the late 1800s was that people were consuming food from a larger geographical area (Saikat et al. 2004). Greater quantities of food grown on allotments (a small portion of usually public land made available for low-cost rental to allow individual food cultivation) could be of concern because of their often urban nature and greater risk of contaminated soil from earlier industrial use or atmospheric deposition (Papritz and Reichard 2009). However, a recent U.K. survey of 12 metals in allotment-grown foods found that levels were generally low (Weeks et al. 2007).

*Impacts on food sourcing and consumption*. Climate change is expected to lead to shifting food belts, which implies that food consumed in the future will be sourced from different parts of the world (Easterling et al. 2007). The source of food may affect its micronutrient and macronutrient composition because of different varieties grown, varying soils and growing conditions, differing methods of harvesting, processing, and storing methods. An example of how geographical sourcing can affect food composition is the element selenium, which may be protective against several types of cancer (World Cancer Research Fund/American Institute for Cancer Research 2007). The U.K. population obtains much of its dietary selenium from grain. From 1970 to 2000, there was a 50% reduction in U.K. dietary selenium intake (Adams et al. 2002) coinciding with a shift in grain importation from Canada to production in the relatively selenium-poor soils of the United Kingdom. There is evidence that daily selenium intakes in the United Kingdom are below recommended levels (Finley 2007). In addition, climate change–induced shifts in where food is produced will alter food safety risks. For example, food from the tropics carries an elevated risk of mycotoxin exposure, and the country of origin may affect microbial risks because of varying policies on the use of wastewater for irrigation (Drechsel et al. 2009).

Climate affects human behavior, and so in an altered climate, individuals may choose to consume different foods. This could have important consequences for nutrition and food safety. For example, U.K. summers are likely to become warmer, and Mintel (2003a, 2003b) showed that the consumption of salad and alcohol is higher in warmer summers than in cooler ones. Few studies have examined how weather affects food consumption, making it difficult to estimate the impact of climate change on diets.

*Adaptation to climate change.* The previous section highlights mechanisms through which climate change could affect the nutritional composition of diets and the safety of food. Whether these changes occur will depend upon local policies and structures to regulate food production, monitor the quality and safety of food, and record and respond to any nutritional or safety issues that arise. Such structures provide a country with the capacity to adapt to climate change. In the next section, we provide an overview of these structures in developed countries using the United Kingdom as a case study. We also discuss how these structures may be enhanced to facilitate adaptation to climate change.

*Nutritional adaptation*. If climate change leads to changes in the nutritional composition of individual diets, then the overall effects will depend upon the ability of society to adapt to these changes. Regular monitoring of the nutritional composition of staples such as grain and potatoes, meat, fruits, and vegetables does not occur in the United Kingdom, but food intake and the nutritional status of the population is monitored through the National Diet and Nutrition Survey (Ashwell et al. 2006), which measures food and nutrient intake and nutritional status of a stratified sample of the UK’s population every 10 years. Although this survey exemplifies good practice in nutritional assessment of a population, it has limitations, and good biomarkers of nutritional status for more vitamins and minerals are urgently needed (Fairweather-Tait 2008). As some effects of climate change upon nutrition may be localized or only affect specific subgroups of the population, there is a need for more targeted monitoring of vulnerable populations such as low-income individuals who are most likely to be affected by rises in food price, those already at nutritional risk (e.g., children, frail elderly), and consumers who choose a diet predominantly sourced from a small geographical area.

If climate change alters the nutritional composition of individual diets, and if these changes are identified, then the overall effect will depend upon local policy responses. Policy responses to existing nutritional issues provide evidence of developed countries’ capacity, using our case study of the United Kingdom, to adapt to nutritional changes associated with climate change. One strategy to address changing nutritional statuses of the population would be the fortification of foods within agriculture (biofortification) or during food processing. For example, white flour is fortified with a variety of minerals and vitamins in the United Kingdom. In addition to fortification, governments may encourage manufacturers to alter the constituents of their food products in response to health concerns. One example is the initiative to reduce the salt content of processed foods (FSA 2009b) where policy in the United Kingdom appears to have reduced salt intake by 10% (FSA 2010). However, such initiatives can face significant opposition from industry (Pendrous 2009).

**Figure 2:**
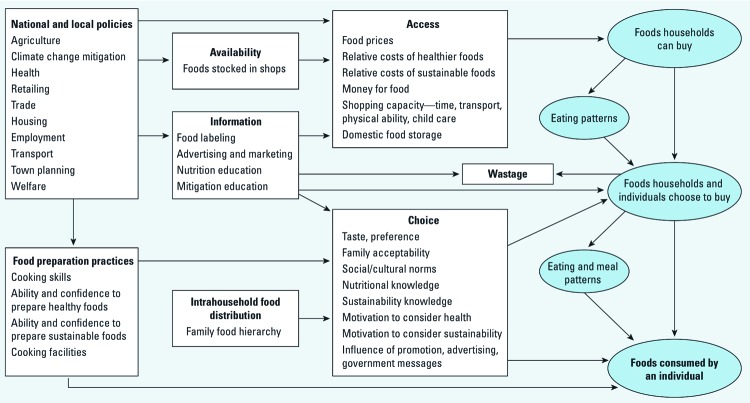
Framework of the determinants of food choice in the United Kingdom. Adapted from Dowler et al. (2001).

Another way to address the issues of climate change–related nutritional status is through altering food intakes. However, this is challenging, as multiple factors affect food choice (Dowler et al. 2001; Figure 2). Simple interventions such as public education campaigns have limited success, especially when they are in direct competition with the marketing of highly processed and flavored foods (ESRC 2008). Targeted interventions such as the Buywell project (described in ESRC 2008) have had better success through use of targeted direct-mail price promotions of healthier products in combination with messages promoting the benefits of dietary change.

*Food safety adaptation*. The permitted levels of many contaminants (microbial, chemical, and radiation) in EU food and many countries (such as the United Kingdom) are established on an international basis through the FAO/WHO (2006) Codex Alimentarius Commission. Therefore, if climate change led to increases above the established levels for food contaminants, such food would not be permitted to enter the human food chain. Some food retailers ensure that their suppliers adhere to limits lower than the regulatory limits (Asfaw et al. 2010). The processes permitted within agriculture and food processing are also strictly controlled to ensure food safety. Examples include the EU Food Hygiene Regulations (European Parliament and the Council of the European Union 2004) and the EU Plant Protection Products Regulations (European Parliament and the Council of the European Union 1991). Standards and regulations have the capacity to prevent food and safety issues resulting from climate change.

To ensure the success of these regulations, food monitoring is required. For example, in the United Kingdom regular food surveys are undertaken by the FSA (2012), and additional surveys are undertaken by other organizations (e.g., Pesticides Residues Committee, London, UK) and individual retailers (Asfaw et al. 2010). In the case of *Campylobacter* levels in poultry, surveys have been used as a basis on which voluntary targets have been established with industry to reduce levels in food further (FSA 2009b). As food surveys can only test a small proportion of foods (because of logistical and budgetary constraints), localized food safety issues are unlikely to be uncovered by national food surveys. This limitation highlights the need for risk assessment along the food chain to identify areas undergoing significant environmental change or rapid agricultural adaptations. Food from such areas would be a target for enhanced monitoring.

Developed countries such as the United Kingdom have disease surveillance structures in place to monitor human illnesses that may result from food contamination. In the United Kingdom, this surveillance is predominantly the responsibility of the Health Policy Agency (HPA). If an outbreak is detected, action is then taken to identify and control the source. In addition, the HPA is involved in monitoring long-term trends in infections. This information has been used to support measures to protect public health. For example, in the United Kingdom the official report on the Stanley Royd outbreak of *Salmonella typhimurium* in 1984, which resulted in 19 deaths (Hugill 1986), led to food safety improvements. If foodborne outbreaks are detected or abnormalities identified through food monitoring, then food chain traceability is essential to identify the source of contamination. The EU General Food Law Regulation contains requirements for food chain traceability (Szajkowska 2009).

Climate change may alter the status quo and thus render current regulation and monitoring of the food chain inadequate and highlights the need for emerging risk identification systems (Marvin et al. 2009) that detect food safety problems at the earliest opportunity. Such techniques include horizon scanning, a method that looks at foodborne diseases emerging in other parts of the world or diseases emerging in animals to predict future threats to humans. The HPA Microbial Risk Assessment Group (HPA 2012) uses horizon scanning to identify and assess threats posed by new or re-emerging infectious diseases. Other options are early warning systems, and the best examples of these are for mycotoxins in maize. These systems use computer models to predict mycotoxin risk using information on current weather conditions (Marvin et al. 2009).

*Conclusions and recommendations*. In the future, food systems are likely to change for a number of reasons, including increased global affluence and the challenges of feeding a global population that may reach nine billion persons by the 2050s (Royal Society 2009). Our review highlights anthropogenic climate change as a further important factor and summarizes some of the impacts that it may have on nutrition and food safety in developed countries. One of the more certain impacts is increasing food prices once global temperatures rise more than 3°C, which may lead to increasingly unhealthy food choices and exacerbate existing health inequalities.

An altered climate will mean that food will be produced under different environmental conditions and, coupled with adaptations to and mitigations against climate change, food production will be very different in the future. These changes will result in emerging pathogens, new crop and livestock species, altered use of pesticides and veterinary medicines and will likely affect the main transfer mechanisms though which contaminants move from the environment to food. All these may have implications for food safety and the nutritional content of food.

Effects of climate change on food safety may be highly localized, with the foods most at risk being those produced in areas undergoing rapid environmental change, agricultural adaptation, or mitigation. Individuals from vulnerable groups where dietary intakes are already suboptimal (e.g., persons with low incomes, migrant workers) and nutrient density requirements are elevated (e.g., pregnancy, childhood, old age) also may be at increased risk. As mitigation against climate change, individuals may start to consume food produced with lower-GHG emissions. Such changes imply lower red meat and dairy consumption, which would have positive effects in terms of lower rates of cardiovascular disease but may result in higher prevalence of iron and zinc deficiencies. Consumption of more locally produced and seasonal food may lead to insufficient fresh fruit and vegetable intakes at various times of the year in temperate countries. Developed countries have monitoring structures and policies that may limit potential effects of climate change on food safety. We suggest that the structures in place to respond to nutritional challenges are less robust, especially due to the potential conflicts between public health and industry.

Much of the climate change and food research discussed in this paper is based on a range of standard IPCC scenarios on how climate may change and has not considered outlier scenarios, changes in extreme events, or more rapid or complex changes in climate (Butler 2010). These conditions could have more drastic consequences for food than those discussed in this paper. However, one of the first assessments of such impacts suggests that a collapse in the Atlantic thermohaline circulation would not have large impacts upon agriculture in Europe (Kuhlbrodt et al. 2009).

Given the significant uncertainty about potential effects of climate change on food security, we recommend further research to quantify possible impacts on nutrition and food safety, including effects resulting from increasing food prices and changes in consumer behavior. In addition, it is important to maintain and strengthen existing structures and policies to regulate food production, monitor the quality and safety of food, and respond to nutritional or safety issues that arise. Climate change also may require enhanced use of emerging risk identification systems to detect new food safety problems at the earliest opportunity. Environmental and health sectors must work together to take advantage of areas of common ground (e.g., promoting reduced red meat consumption to lower GHG emissions and reduce the incidence of ischemic heart disease) and resolve potential conflicts (e.g., greater consumption of seasonal food to lower GHG emissions conflicting with health goals for year round consumption of fruit and vegetables). Such cooperation is essential to provide consistent health and environmental messages to the public and develop suitable interventions.

## References

[r1] Adak GK, Meakins SM, Yip H, Lopman BA, O’Brien SJ (2005). Disease risks from foods, England and Wales, 1996–2000.. Emerging Infect Dis.

[r2] Adams ML, Lombi E, Zhao FJ, McGrath SP (2002). Evidence of low selenium concentrations in UK bread-making wheat grain.. J Sci Food Agric.

[r3] ADAS (Agricultural Land Advisory Service) (2009). RMP/5142 Analysis of Policy Instruments for Reducing Greenhouse Gas Emissions from Agriculture, Forestry and Land Management.

[r4] Asfaw S, Mithöfer D, Waibel H (2010). What impact are EU supermarket standards having on developing countries’ export of high-value horticultural products? Evidence from Kenya.. J Int Food Agribusiness Market.

[r5] Ashwell M, Barlow S, Gibson S, Harris C (2006). National diet and nutrition surveys: the British experience.. Public Health Nutr.

[r6] Banse M, Van Meijl H, Tabeau A, Woltjer G (2008). Will EU biofuel policies affect global agricultural markets?. Eur Rev Agricult Econ.

[r7] Barrett J, Vallack H, Jones A, Haq G (2002). A Material Flow Analysis and Ecological Footprint of York: Technical Report.

[r8] Battisti DS, Naylor RL (2009). Historical warnings of future food insecurity with unprecedented seasonal heat.. Science.

[r9] Blanke MM, Burdick M (2005). Food (miles) for thought—energy balance for locally-grown versus imported apples fruit.. Environ Sci Pollut Res Int.

[r10] Boxall ABA, Hardy A, Beulke S, Boucard T, Burgin L, Falloon PD (2009). Impacts of climate change on indirect human exposure to pathogens and chemicals from agriculture.. Environ Health Perspect.

[r11] Butler CD (2010). Climate change, crop yields, and the future.. SCN News.

[r12] Chen C-C, McCarl BA (2001). Pesticide usage as influenced by climate: a statistical investigation.. Clim Change.

[r13] Cohen MJ, Tirado C, Aberman N-L, Thompson B (2008). Impact of Climate Change and Bioenergy on Nutrition.

[r14] Cooper S, Nelson M (2003). ‘Economy’ line foods from four supermarkets and brand name equivalents: a comparison of their nutrient contents and costs.. J Hum Nutr Diet.

[r15] Cummins S, Macintyre S (2006). Food environments and obesity—neighbourhood or nation?. Int J Epidemiol.

[r16] Dangour AD, Dodhia SK, Hayter A, Allen E, Lock K, Uauy R (2009). Nutritional quality of organic foods: a systematic review.. Am J Clin Nutr.

[r17] Defra (Department for Environment, Food and Rural Affairs) (2011a). Evidence to Define the Sustainability of a Healthy Diet—FO0430. Annex A: Environmental Sustainability.

[r18] Defra (Department for Environment, Food and Rural Affairs) (2011b). New Diets for Cows Could Cut Climate Emissions.. http://www.defra.gov.uk/news/2011/04/01/cow-emissions/.

[r19] Döll P (2002). Impact of climate change and variability on irrigation requirements: a global perspective.. Clim Change.

[r20] Dowler EA, Turner S, Dobson BM (2001). Poverty Bites: Food, Health and Poor Families.

[r21] Drechsel P, Scott CA, Raschid-Sally L, Redwood M, Bahri A, eds (2009). Wastewater Irrigation and Health. Assessing and Mitigating Risk in Low-income Countries.

[r22] Easterling WE, Aggarwal PK, Batima P, Brander KM, Erda L, Howden SM (2007). Food, fibre and forest products. In: Climate Change 2007: Impacts, Adaptation and Vulnerability Contribution of Working Group II to the Fourth Assessment Report of the Intergovernmental Panel on Climate Change (Parry ML, Canziani OF, Palutikof JP, van der Linden PJ, Hanson CE, eds).

[r23] EEA (European Environment Agency) (2007). Europe’s Environment—The Fourth Assessment.

[r24] Embase (2009). Embase Quick Search.. http://www.embase.com/.

[r25] ESRC (Economic and Social Research Council) (2008). ESRC Seminar Series: Mapping the Public Policy Landscape. Public Behaviour in the UK in Times of Economic Decline/Rising Food Prices.. Swindon, UK:ESRC.

[r26] European Parliament and the Council of the European Economic Commission (1991). Regulation (EC) No. 91/414/EEC of 19 August 1991 concerning the placing of plant protection products on the market.. Off J Eur Union.

[r27] European Parliament and the Council of the European Union (2004). Regulation (EC) No. 852/2004 of 29 April 2004 on the hygiene of foodstuffs.. Off J Eur Union.

[r28] Fairweather-Tait SJ 2008 Biomarkers of micronutrient status. Br J Nutr 99 S1 doi:10.1017/S0007114508006788 [Online 4 July 2008]

[r29] Fan M-S, Zhao F-J, Fairweather-Tait SJ, Poulton PR, Dunham SJ, McGrath SP (2008). Evidence of decreasing mineral density in wheat grain over the last 160 years.. J Trace Elem Med Biol.

[r30] FAO (Food and Agriculture Organization of the United Nations) (2008). Climate Change: Implications for Food Safety.

[r31] FAO/WHO (Food and Agriculture Organization of the United Nations and the World Health Organizaton) (2006). Understanding the Codex Alimentarius. Third Edition.

[r32] Finley JW (2007). Increased intakes of selenium-enriched foods may benefit human health.. J Sci Food Agric.

[r33] Foster R, Lunn J (2007). 40th anniversary briefing paper: food availability and our changing diet.. Nutr Bull.

[r34] Friel S, Dangour AD, Garnett T, Lock K, Chalabi Z, Roberts I (2009). Public health benefits of strategies to reduce greenhouse-gas emissions: food and agriculture.. Lancet.

[r35] FSA (Food Standards Agency) (2009a). A UK Survey of *Campylobacter* and *Salmonella* Contamination of Fresh Chicken at Retail Sale.

[r36] FSA (Food Standards Agency) (2009b). Salt Reduction Strategy.. http://collections.europarchive.org/tna/20100927130941/http://food.gov.uk/healthiereating/salt/strategy.

[r37] FSA (Food Standards Agency) (2010). WHO Salt Reduction Talks.. http://www.food.gov.uk/news/speeches/timsmithspeeches/whosaltreductiontalks.

[r38] FSA (Food Standards Agency) (2012). Food Surveys Published in 2011.. http://www.food.gov.uk/science/research/surveillance/fsisbranch2011/#.UF9dN1FrX4w.

[r39] Garnett T (2006). Fruit and Vegetables and UK Greenhouse Gas Emissions: Exploring the Relationship. Food Climate Research Network.

[r40] Garnett T (2008). Cooking Up A Storm. Food, Greenhouse Gas Emissions and Our Changing Climate.

[r41] Google (2009). Google UK Homepage.. http://www.google.co.uk.

[r42] Harrus S, Baneth G (2005). Drivers for the emergence and re-emergence of vector-borne protozoal and bacterial diseases.. Int J Parasitol.

[r43] Hegerl G, Luterbacher J, González-Rouco F, Tett SFB, Crowley T, Xoplaki E (2011). Influence of human and natural forcing on European seasonal temperatures.. Nat Geosci.

[r44] HPA (Health Protection Agency) (2012). Microbial Risk Assessment Group.. http://www.hpa.org.uk/web/HPAweb&HPAwebStandard/HPAweb_C/1195733849356.

[r45] Hugill J (1986). The Report of the Committee of Inquiry into an Outbreak of Food Poisoning at Stanley Royd Hospital, Cmnd 9716.

[r46] IPCC (Intergovernmental Panel on Climate Change) (2007). Climate Change 2007: Synthesis Report. Contribution of Working Groups I, II and III to the Fourth Assessment Report of the Intergovernmental Panel on Climate Change (Core Writing Team, Pachauri RK, Reisinger A, eds.).

[r47] ISI Web of Knowledge (2009). Web of Knowledge Service for UK Education Homepage.. http://wok.mimas.ac.uk/.

[r48] Jungk J, Baumbach J, Landen M, Gaul LK, Alaniz L, Dang T (2008). Outbreak of *Salmonella* serotype Saintpaul infections associated with multiple raw produce items—United States, 2008.. MMWR Morb Mortal Wkly Rep.

[r49] Kemper N (2008). Veterinary antibiotics in the aquatic and terrestrial environment.. Ecol Indicators.

[r50] Kovats RS, Edwards SJ, Charron D, Cowden J, D’Souza RM, Ebi KL (2005). Climate variability and campylobacter infection: an international study.. Int J Biometeorol.

[r51] Kovats RS, Edwards SJ, Hajat S, Armstrong BG, Ebi KL, Menne B (2004). The effect of temperature on food poisoning: a time-series analysis of salmonellosis in ten European countries.. Epidemiol Infect.

[r52] Kuhlbrodt T, Rahmstorf S, Zickfeld K, Vikebø FB, Sundby S, Hofmann M (2009). An integrated assessment of changes in the thermohaline circulation.. Clim Change.

[r53] Lake IR, Gillespie IA, Bentham G, Nichols GL, Lane C, Adak GK (2009). A re-evaluation of the impact of temperature and climate change on foodborne illness.. Epidemiol Infect.

[r54] Lobell DB, Schlenker W, Costa-Roberts J (2011). Climate trends and global crop production since 1980.. Science.

[r55] Lock K Stuckler D Charlesworth K McKee M 2009 Potential causes and health effects of rising global food prices. BMJ 339 b2403 doi:10.1136/bmj.b2403 [Online 13 July 2009] 19596718

[r56] MacGregor J, Vorley B, eds (2006). Fresh Insights Number 9. Fair Miles? Weighing Environmental and Social Impacts of Fresh Produce Exports from Sub-Saharan Africa to the UK.

[r57] Marvin HJP, Kleter GA, Prandini A, Dekkers S, Bolton DJ (2009). Early identification systems for emerging foodborne hazards.. Food Chem Toxicol.

[r58] McMichael AJ, Woodruff RE, Hales S (2006). Climate change and human health: present and future risks.. Lancet.

[r59] Mintel (2003a). Drinks Market—UK—November 2003.

[r60] Mintel (2003b). Pre-packed and Dressed Salads—UK—October 2003.

[r61] National Library of Medicine (2009). MEDLINE/PubMed Resources Guide.. http://www.nlm.nih.gov/bsd/pmresources.html.

[r62] Oliver MA (1997). Soil and human health: a review.. Eur J Soil Sci.

[r63] Papritz A, Reichard PU (2009). Modelling the risk of Pb and PAH intervention value exceedance in allotment soils by robust logistic regression.. Environ Pollut.

[r64] Pendrous R (2009). It’s Time for FSA Salt Reduction Targets to Take a Reality Check.. http://www.foodmanufacture.co.uk/Business-News/It-s-time-for-FSA-salt-reduction-targets-to-take-a-reality-check.

[r65] Piesse J, Thirtle C (2009). Three bubbles and a panic: an explanatory review of recent food commodity price events.. Food Policy.

[r66] Plumlee GS, Foreman WT, Griffin DW, Lovelace JK, Meeker GP, Demas CR (2007). Characterization of flood sediments from hurricanes Katrina and Rita and potential implications for human health and the environment. In: Science and the Storms: The USGS Response to the Hurricanes of 2005. Circular 1306 (Farris GS, Smith GM, Crane MP, Demas CR, Robbins LL, Lavoie DL, eds).

[r67] Royal Society (2009). Reaping the Benefits: Science and the Sustainable Intensification of Global Agriculture.

[r68] Rural and Environment Analytical Services (2009). Food Prices: An Overview of Current Evidence.

[r69] Russell R, Paterson M, Lima N (2010). How will climate change affect mycotoxins in food?. Food Res Int.

[r70] SACN (Scientific Advisory Committee on Nutrition) (2006). Paper for Agreement: NDNS Synthesis Paper. Agenda Item: 4.

[r71] SACN (Scientific Advisory Committee on Nutrition) (2010). Iron and Health.

[r72] Saikat SQ, Carter JE, Mehra A, Smith B, Stewart A (2004). Goitre and environmental iodine deficiency in the UK—Derbyshire: a review.. Environ Geochem Health.

[r73] SCOPUS (2009). Document Search Homepage.. http://www.scopus.com.

[r74] Smith A, Watkiss P, Tweddle G, McKinnon A, Browne M, Hunt A (2005). The Validity of Food Miles as an Indicator of Sustainable Development: Final Report for DEFRA.

[r75] Stern N (2006). The Economics of Climate Change: The Stern Review.

[r76] Strategy Unit (2008). Food Matters: Towards a Strategy for the 21st Century.

[r77] Szajkowska A (2009). From mutual recognition to mutual scientific opinion? Constitutional framework for risk analysis in EU food safety law.. Food Policy.

[r78] Tirado MC, Clarke R, Jaykus LA, McQuatters-Gollop A, Frank JM (2010). Climate change and food safety: a review.. Food Res Int.

[r79] United Kingdom Climate Impacts Programme (2011). Thinking Climate—Putting Scientific Knowledge into the Heart of Decision-making.. http://www.ukcip.org.uk/index.php.

[r80] Wallén A, Brandt N, Wennersten R (2004). Does the Swedish consumer’s choice of food influence greenhouse gas emissions?. Environ Sci Policy.

[r81] Weeks CA, Brown SN, Vazquez I, Thomas K, Baxter M, Warriss PD (2007). Multi-element survey of allotment produce and soil in the UK.. Food Addit Contam.

[r82] WHO (World Health Organization) (1990). Diet, Nutrition, and the Prevention of Chronic Diseases.

[r83] WHO (World Health Organization) (2006). WHO Guidelines for the Safe Use of Wastewater, Excreta and Greywater. Volume II: Wastewater Use in Agriculture.

[r84] WHO (World Health Organization) (2008). Worldwide Prevalence of Anaemia 1993–2005: WHO Global Database on Anaemia.

[r85] WHO (World Health Organization) (2010). Trade, Foreign Policy, Diplomacy and Health. Food Security.. http://www.who.int/trade/glossary/story028/en/.

[r86] World Cancer Research Fund/American Institute for Cancer Research (2007). Food, Nutrition, Physical Activity, and the Prevention of Cancer: A Global Perspective.

